# What Entrepreneurial Followers at the Start-Up Stage Need From Entrepreneurship Cultivation: Evidence From Western China

**DOI:** 10.3389/fpsyg.2019.01309

**Published:** 2019-06-04

**Authors:** Xuefan Dong, Chengxiang Tang, Ying Lian, Daisheng Tang

**Affiliations:** ^1^School of Economics and Management, Beijing Jiaotong University, Beijing, China; ^2^School of Public Administration, Guangzhou University, Guangzhou, China

**Keywords:** entrepreneurship, entrepreneurial follower, entrepreneurship cultivation, start-up stage, Western China

## Abstract

Entrepreneurial followers are defined as the crucial members of a specific entrepreneurial team and do not include the leader or normal employees in the present paper. This population can be viewed as indispensable factors in the success of entrepreneurship, especially in the start-up stage. In addition, according to the following time, they can be divided into two groups, namely long-term entrepreneurial followers and short-term entrepreneurial followers. However, studies focusing on entrepreneurship cultivation for entrepreneurial followers are relatively few. The main purpose of this paper is to determine the needs of Chinese entrepreneurial followers in entrepreneurship cultivation from the early stage of entrepreneurship. In this paper, a sample of 200 long-term entrepreneurial followers from Tianfu New Area in China was investigated. To enable the researchers to explore the unique opinions of entrepreneurial followers, a mixed data collection approach that combined interviews and questionnaires was chosen in this study. The results revealed following findings: (a) high levels of social capital, good entrepreneurial opportunities and projects, and highly cooperative teams were viewed as the most important factors for entrepreneurship by entrepreneurial followers in China; (b) most entrepreneurial followers believed that the primary difficulty in the cultivation process was the inefficiency in talent training mechanism; and (c) nearly 40% of samples suggested that the cultivation and enhancement of local talents should be firstly carried out by the Chinese government, indicating a gap between the supporting force for local and returned talents in China. In addition, various types of incentive policies and good environments for talent growth were also considered as important suggestions by entrepreneurial followers. We found that unlike entrepreneurial leaders, entrepreneurial followers focus more on income expectation, and personal development rather than supporting the development of companies in China. These findings should be viewed as priorities when enhancing current entrepreneurship cultivation in China.

## Introduction

Entrepreneurship has attracted the attention of policymakers, economists, and scholars around the world. In general, entrepreneurship can be defined as an employment opportunity enabling individuals to be self-employed (Matlay, 2013). During the whole process of entrepreneurship, the problem in the start-up stage is the biggest obstacle facing entrepreneurs (Raposo and Do, 2011), because start-up companies have only two options: either it fails, and it dies (Villéger, 2018). Previous studies have confirmed the important role played by entrepreneurs in entrepreneurship (Shane and Venkataraman, 2000). This increased interest has led to a growing body of research that has attempted to identify the factors of entrepreneurs that can be promoted by its cultivation. It is generally accepted by policymakers and scholars that high levels of entrepreneurship can be achieved through cultivation. This assumption proves that entrepreneurs’ abilities can be cultivated and are not fixed personal features (Oosterbeek et al., 2010). However, as far as our knowledge goes, studies regarding the cultivation of entrepreneurs only considered the financial (Elston and Audretsch, 2010; Román et al., 2013) and political (Peng et al., 2014) aspects. Thus, they could not provide a comprehensive investigation that focused on the real situations of entrepreneurs during start-up stage, the most difficult and momentous period. Additionally, as Pittaway and Cope (2016) pointed out, the empirical evidence does not often offer conclusive support in this field.

Moreover, existing studies also suggested that, despite the important role played by the entrepreneurial leader as a lone hero, their followers also hold influence in promoting the development of entrepreneurship, especially in the early stages (Ensley et al., 2006; Patterson et al., 2008). However, the entrepreneurial follower is not clearly defined yet. In most previous studies related to the leadership area where leader-follower relationship is frequently discussed, the followers are simply regarded as normal employees and only little recent attention has been paid to followership (Ilies et al., 2005; Jensen and Luthans, 2006; Uhl-Bien et al., 2014). As Uhl-Bien et al. (2014) stated that, the main cause for this shortcoming refers to the confusion and misunderstanding about the definition of followership or followers. This problem is even more serious in entrepreneurship research. Although many scholars have implemented empirical studies on various related issues and provided some evidence indirectly supporting the importance of entrepreneurial followers (Beckman and Burton, 2008; Klotz et al., 2014; Jin et al., 2016; Forsström-Tuominen et al., 2017), how to accurately define this special group is still not considered.

Thus, in order to fill this theoretical gap that currently exists in the literature, the present paper aims to propose the definition of entrepreneurial followers. We argued that entrepreneurial followers could be defined as the crucial members (or crucial employees) of a specific entrepreneurial team and do not include the leader or normal employees. They have highly consistent interests or beliefs with leaders and are generally responsible for different important components of entrepreneurial activities. Instead of being simply viewed as the people being led, entrepreneurial followers are precious human resources for entrepreneurship with characteristics of initiative, activeness, enthusiasm, and creativity.

In addition, only a little evidence has been proposed to support the view that there are considerable differences in personal characteristics and expectations between entrepreneurial leaders and followers. As an example, Ensley et al. (2006) stated that compared to the leaders, followers are more likely to lack confidence in their innovation achievements in uncertain environments. Therefore, by regarding 1 of 19 new state-level areas in China that were established upon approval of the State Council—namely, the Tianfu New Area, located in Sichuan Province, this investigation focuses on answering the following question: What do entrepreneurial followers really need in entrepreneurship cultivation at the start-up stage? In total, 3 aspects will be considered in relation to this question: (a) The factors that influence entrepreneurial followers in participating in entrepreneurship activities; (b) the difficulties faced by entrepreneurial followers in early stage entrepreneurship; and (c) suggestions for the improvement of the system of entrepreneurship cultivation at the start-up stage. By taking these three aspects into considerations from entrepreneurial followers’ own perspectives, a more comprehensive and precise investigation regarding the cultivation of them at the start-up stage could be provided. Thus, this paper has the potential to address the existing gap in entrepreneurship cultivation, which has been neglected by previous studies.

The present paper is organized as follows. The next section provides a literature review about the topic. The section “Materials and Methods” demonstrates the applied method of this paper. The section “Results” presents the empirical results. The section “Conclusion and Discussion” draw on the main conclusion and implications extracted from the results.

## Related Works

Previous studies indicated that the entrepreneurship is important for social, national, and industrial development (Shane and Venkataraman, 2000; Raposo and Do, 2011; Zhang et al., 2014). For instance, Zhang et al. (2014) stated that entrepreneurship contributes to the incubation of technological innovation, increases economic efficiency, and creates new jobs. Raposo and Do (2011) argued that the improvement of innovation performance and the well-being of citizens are also considerable factors that contributed to a high level of entrepreneurial activity. Shane and Venkataraman (2000) suggested that entrepreneurship is a good way of showing new technical information that is embodied in products and services. In addition, various aspects such as age (Bohlmann et al., 2017), gender (Zampetakis et al., 2017), personality (Vries, 2010; Hu et al., 2018), cognitive style (Kickul et al., 2010), decision-making abilities of entrepreneurs (Liu, 2018), and the optimization of the allocation of entrepreneurial resources (Dunkelberg et al., 2013) are recent research topics found in the literature.

Much evidence to support that entrepreneurship capabilities can be cultivated and are not fixed personal features has been published in existing research. For instance, Karimi et al. (2016) stated that effective cultivation can foster entrepreneurial competences. In addition, as Sánchez (2011), Chia (2010), and Piperopoulos and Dimov (2014) suggested, despite the knowledge and skills necessary to begin and run a business, the improvement of certain beliefs, values, and attitudes is the main achievement of entrepreneurial cultivation. With respect to these improvements from a cultivation perspective, the governmental influence is frequently considered, especially in relation to educational and industrial policies (Verheul et al., 2002; Sánchez, 2011). Raposo and Do (2011) claimed that policy can affect entrepreneurship in two ways: directly, through special measures, and indirectly, through generic measures. Pittaway and Cope (2016) cited political issues as the first macro level of entrepreneurship cultivation, and they believed that the second macro level should refer to general enterprise infrastructure. To be more specific, these themes can be viewed as the input and output of the domain of entrepreneurship cultivation, respectively.

From a framework perspective, Jamieson (1984) divided entrepreneurship cultivation into 3 categories by considering different types of cultivation—namely, cultivation for enterprise, which aims to educate students on awareness creation from a theoretical perspective; cultivation in enterprise, which focuses on encouraging participants to begin their own business; and the cultivation in enterprise that includes but is not limited to training management and business expansion. The first category refers to the level of universities or higher-learning institutions that have attracted the attention of the majority of researchers who focus on entrepreneurship cultivation (e.g., Saboe et al., 2002; Rindova et al., 2012; Galloway and Brown, 2013; Küttim et al., 2014; Din et al., 2016). Such a high level of research interest is mainly due to the general consensus that youths are the most important participants in entrepreneurship; there is a significantly positive relationship between the effectiveness of education programs in universities and learning institutions and youths’ intentions of entrepreneurship. Din et al. (2016) stated that many graduates prefer to find positions in public and private sectors with high levels of competitiveness and income rather than becoming entrepreneurs due to their lack of knowledge, awareness, and skills. This is primarily owing to the deficiency of entrepreneurship educational programs in universities. Moreover, some scholars even recommend that entrepreneurship education should be implemented earlier (Sexton and Landström, 2000). In addition, with respect to the third category, the educated population are often small-business owners who have achieved some success. In other words, the point of concern in this category is not whether the entrepreneurs could start a business, but how to run the business more successfully. Therefore, exiting studies related to this aspect could be classified into the area of small-business management (Gorman et al., 1997; Neck and Greene, 2011). However, despite the inspiration that was cultivated in universities and learning institutions and the management capabilities gained in enterprise-training activities, how to carry out the practice in reality is a considerably important point to be discussed, especially at the start-up stage. According to Raposo and Do (2011), this is a typical example of the second category, which refers to the hardest time in the entrepreneurship. In general, start-up companies simply refer to young innovative companies (Zaech and Baldegger, 2017). In this view, the age of companies has been proved to be a widely accepted assessment criterion to judge whether a company is going through the start-up stage (Pellegrino et al., 2012). According to Villéger (2018), the maximum age to define start-up companies varies from 5 to 12 years. Additionally, growth, organizational flexibility, and limited human and finical resources are also typical characteristics of start-up companies that have been identified by existing research (Liao and Welsch, 2003; Peterson et al., 2009).

In addition to the temporal perspective, many scholars have researched entrepreneurship cultivation from a spatial point of view, in which the United States (Worsham and Dees, 2012; Elert et al., 2015; Guo et al., 2016) and the United Kingdom (Matlay, 2009; Henry and Treanor, 2010; Dabic et al., 2016) are frequently discussed. In addition, other developed European countries, such as France (Klapper, 2004; Kövesi, 2017), Germany (Klandt, 2004), and Sweden (Dahlstedt and Fejes, 2017) have also been targeted by existing studies. However, a focus on developing countries, such as China, is relatively low in English-language literature, and if developing countries were discussed, it was mostly in consideration of university students only. For instance, Wu and Wu (2008) investigated the relationship between Chinese university students’ higher educational backgrounds and their entrepreneurial intentions. Zhou and Xu (2012) evaluated the state of entrepreneurship education from a student level in China and compared it to the United States. Li et al. (2013) discussed the critical factors in Chinese higher-educational institutions that may shape the directions of entrepreneurship education. Tian et al. (2016) conducted a knowledge map for studies related to entrepreneurship education in China from 2004 to 2013. Xu et al. (2016) reviewed entrepreneurship education in Chinese secondary schools. It is interesting to note that China did not begin any entrepreneurship education programs until 2002, when the Ministry of Education published a pilot project for entrepreneurship education, and the effectiveness of this project is considerable. According to a global entrepreneurship survey, China jumped from eleventh place in 2002 to second place in 2012 in the entrepreneurship composite index rankings of more than 60 countries and regions (Li et al., 2013). Therefore, more in-depth research that targets the development process and current state of Chinese entrepreneurship cultivation is of great significance from a global perspective. Furthermore, as mentioned above, a comprehensive investigation should be implemented, with empirical evidence used to identify useful educational factors that ensure success for entrepreneurs at the start-up stage.

In the view of entrepreneurial followers, some scholars have implemented empirical studies that focus on various related issues (Beckman and Burton, 2008; Jin et al., 2016; Forsström-Tuominen et al., 2017). For instance, Jin et al. (2016) conducted a meta-analysis to investigate the relationship between the composition features of entrepreneurial teams and new venture performance. New venture performance can generally be defined as the development and growth of companies at the start-up stage (Klotz et al., 2014). Jin et al. (2016) suggested that the individual ability of entrepreneurial followers could contribute to a higher level of new venture performance. Additionally, Forsström-Tuominen et al. (2017) applied a qualitative multiple-case study to analyze the initiation and formation of entrepreneurial teams in the start-up stage, based on individual- and group-interview data from 4 high-tech teams. They found that in addition to economic and technical issues, the various social and psychological aspects, such as collective encouragement, could be viewed as another important impetus to initiate an entrepreneurial team. It seems that to a large extent, there would be no entrepreneurship without a team. This argument emphasizes the significant value of entrepreneurial members in early stage entrepreneurship. However, the definition of entrepreneurial followers still remains unclear. Instead, most existing studies mixed entrepreneurial leaders and followers, especially for those focused on the entrepreneurship cultivation (Dabic et al., 2016; Din et al., 2016). It seems that such a practice is overly broad and inevitably leads to a reduction in pertinence. Therefore, the present paper provided the definition of entrepreneurial follower (see the section “Introduction”) in order to identify the main differences between entrepreneurial leaders and followers, and accordingly find the needs of entrepreneurial followers in entrepreneurship cultivation during the start-up stage.

Moreover, according to the following time, we argued that the entrepreneurial followers can be divided into two categories: long-term entrepreneurial followers and short-term entrepreneurial followers. In comparison, the former shows greater loyalty and autonomy, and has more interest or belief foundation with the leaders. In addition, the behaviors of leader are also more likely to be shaped and altered by the former because the long-term closely personal, social and working relationships between them. Many historical examples have proved the importance of long-term entrepreneurial followers for the success of entrepreneurship. As Cooney (2005) stated that, when one considers the success of Apple, Steve Jobs may immediately spring to mind for most people. However, such great success could not be achieved without Steve Wozniak, who invented the model for the first personal computer, or Mike Markkula, who provided access to venture capital. In addition, with respect to the Alibaba Group, a world-famous Chinese company, apart from the personal capacity of Ma Yun, who is the founder and executive chairman, the success at the start-up stage of venturing cannot be separated from the role of his followers, including but not limited to Jianhang Jin, who was responsible for marketing, and Yongming Wu, who provided technical support.

## Materials and Methods

### Study Design

Since the goal of this study is to both “explore” and “measure” the needs of entrepreneurial followers in entrepreneurship cultivation, it not only demands a more qualitative design (Marshall et al., 2015), but also requires a quantitative consideration. Thus, a mixed data collection approach that combined interviews and questionnaires was employed in the present paper to obtain a thorough exploration of the samples’ opinions. According to previous studies, through more than one method to collecting data on the same topic can minimize the weaknesses of quantitative and qualitative methodologies in single research investigations and across investigations (Alaiad and Zhou, 2017). It can also give a greater perspective and understanding for interpreting the quantitative results by containing more supplementary qualitative techniques (Pope and Mays, 1995; Venkatesh et al., 2013). In addition, validity or credibility of evaluation findings can be enhanced and the representativeness of the study can be strengthened by using a mixed data collection approach (Ferreira et al., 2019). For example, by using a mixed method that combined an interview and survey, Alaiad and Zhou (2017) comprehensively identified some important concerns of several healthcare patients and medical professionals regarding the adoption of the WSN-Based Smart Home Healthcare Systems (WSN-SHHS). With respect to our study, the application of this mixed data collection approach by considering both qualitative interviews and quantitative questionnaires could help us obtain rich insights into the phenomena of interest, thus providing a more comprehensive and deeper understanding entrepreneurial followers’ needs in the entrepreneurship cultivation. In specific, employing qualitative interviews can yield broader and more detailed accounts from samples’ own views and beliefs toward the entrepreneurship cultivation that are potentially important to them and allow us to gain access to acquire various underlying mechanisms and reasons behind their perceptions, and the use of quantitative questionnaires was to develop additional more targeted and hierarchical opinions concerning the entrepreneurship cultivation.

### Sampling

Our data source is the Tianfu New Area, Chengdu city, Sichuan province, China. Since ancient times, Chengdu was an important center where entrepreneurs gathered. For example, Bing Li, who was a world-famous hydraulic engineer in the Warring States Period, designed the Dujiangyan Irrigation System. The *jiaozi* (a kind of Chinese ancient currency) was also firstly invented in Chengdu during the time of the Northern Song Dynasty, which created the paper currency system that is still in use today. In addition, the receptive and inclusive culture that is rooted in Sichuan province is currently a fundamental characteristic of the Tianfu New Area. Based on recent developments, officials have planned for the construction of the Luxi Chile Valley Technology Innovation and High-Tech Service Function Area in the Tianfu New Area, and the construction of several new parks, such as the Tianfu Haichuang Park and the Tianfu Science and Technology Park, has already begun. Additionally, it has seen the introduction of key national laboratories such as the Industrial Big Data National Engineering Laboratory, which has welcomed many renowned scientific-research universities and institutions, including but not limited to Tsinghua University, Peking University, Beihang University, and the Chengdu Science Research Center of Chinese Academy of Sciences. Moreover, the Tianfu New Area is the location of many well-known enterprises such as the Chengdu Mengsheng Electronics Limited Company and the China Aerospace Science and Industry Corporation. In recent years, several intellectual property service agencies (represented by Liushen Law Firm) and science and technology financial service agencies (represented by Granite Global Ventures Capital) have successfully settled in the area.

The reason for choosing this area as our data source is because it is 1 of 19 state-level “new” areas in China that was established upon approval of the State Council in October 2014. In 2017, the Tianfu New Area introduced the “Policy Proposals for Accelerating Technological Innovation and Developing High-Tech Services” and the “Implementing Measures for the “Tianfu Elite Program” of the Chengdu Zone of Tianfu New Area.” A special fund has also been established, with 1 billion RMB allocated for talent development. Since its launch in 2014, the Tianfu New Area has attracted more than 1,400 innovative enterprises and 14,600 entrepreneurs, including 12 national experts, 21 provincial experts, 2 expert teams, 24 experts chosen by the Chengdu Talent Planning Program, and 9 foreign experts. In addition, entrepreneurship has developed in these areas in a considerably active way. In addition, there is a large number of entrepreneurial teams at different stages of the entrepreneurship process, thus providing a dedicated support for our investigation. We applied the typical probability sampling method to include companies across various industries in this area. The industries included but were not limited to the technology service industry, the information service industry, the business service industry, the cultural and creative industry, and the high-tech manufacturing industry.

Companies from all seven districts (Chengdu Straight Zone, Chengdu High Tech Zone, Shuangliu, Longquanyi, Xinjin, Jianyang, and Meishan) were considered to ensure that the participants included in this study allowed for optimal generalization. By following this rule, based on the typical probability sampling method, 10 companies were selected. According to the sample, the following six points can be noticed: (a) The registered place of all surveyed companies is in Sichuan province; (b) 73.3% of selected companies are in the creation or seed stages; (c) the registration time of 63.3% of companies is 2015, and the others were registered in 2014; (d) the registered capital of 30% of surveyed companies is below 500,000 RMB, 56.7% is between 1 million RMB (including) and 5 million RMB, and 13.3% is more than 10 million RMB; (e) the number of followers in 53.4% of companies is under 10, and it is between 12 and 45 in 40% of the companies; and (f) the asset value of 40% companies is below 1 million RMB, and 26.7% of companies have an asset value of between 1 and 5 million RMB.

As previously mentioned, entrepreneurial followers refer to the crucial members of an entrepreneurial team, aside from the leader and normal employees. In addition, because the long-term entrepreneurial followers generally play a more important role for the development of companies compared to short-terms ones, they have greater significance to be researched, to a large extent. Thus, in the present paper, only the first-generation entrepreneurial followers of these 10 companies who have followed the leaders for a long period of time (long-term entrepreneurial followers) and made significant contributions to the selected companies were considered.

### Data Collection

By following Alaiad and Zhou (2017), we began with semi-structured interviews to collect entrepreneurial followers’ own perceptions about the entrepreneurial cultivation, and then asked them to select the most appropriate one from the options contained in a structured questionnaire survey (some questions contained in the survey were shown in Table A1 in the Appendix). During the interviewing process, we firstly explained the informed consent at the beginning to make sure that the samples were ensured confidentiality and protection of their privacy. Then, the objective of this study and scenarios to the samples were introduced. In addition, in order to encourage the interviewees to develop contacts and dialogues, a relatively free interview atmosphere was built by beginning with open-ended questions in each interview. A topic guide (see Table A2 in the Appendix) was applied to help interviewers to cover the main points in each interview, and to remain flexible to allow interviewees to introduce different issues of interest to them.

By applying this mixed data collection method, we surveyed and interviewed all of the long-term entrepreneurial followers separately and considered 2 rules for implementing the filtration process of surveys: (a) Ensuring the completeness and validity of the surveys; (b) ensuring industry diversity of the investigated companies. After the data-cleaning procedure, we obtained 200 samples. All of the data obtained from the participants were encrypted to ensure data security.

### Data Analyze

#### Qualitative

Within the process of qualitative data analysis, two researchers (XD and YL) of this paper transformed pronouns and other indexical terms into nominal meanings in order to provide clearer surrounding concepts. Further, these concepts or comments were integrated and categorized based on their central themes by all the researchers of this paper.

#### Quantitative

The collected quantitative data were analyzed using Stata version 14.0. Firstly, the crossover analyses between the entrepreneurship industry and education levels, and between the number of entrepreneurial followers and the annual revenues of the researched companies were carried out. Subsequently, differences in samples’ responses on each question contained in the survey were compared descriptively based on the calculated percentages. The analysis was conducted by XD and YL with review by CT and DT.

### Reliability and Validity Test

The reliability test cannot be carried out in our study because the designed survey does not contain scales. In this view, Gikandi et al. (2011) suggested that a mixed approach combing quantitative and qualitative techniques is useful to assess the degree of reliability in questionnaires. Therefore, we implemented the semi-structured interviews to collect qualitative data, which could ensure the reliability degree of our survey, to a large extent. Additionally, we also carried out a validity test. The results are shown in Table 1. It can be seen that the Kaiser-Meyer-Olkin (KMO) value is larger than 0.5, and the *P* value of Bartlett’s sphericity test is less than 0.05. Thus, the validity of the survey can be guaranteed.

**Table 1:** Validity test results of the survey.

KMO and Bartlett test		
KMO measure of sampling adequacy		0.635
Sphericity test of Bartlett	Approximate chi square	4618.551
	df.	105
	Sig.	0.000


## Results

### Entrepreneurial Followers

**FIGURE 1:**
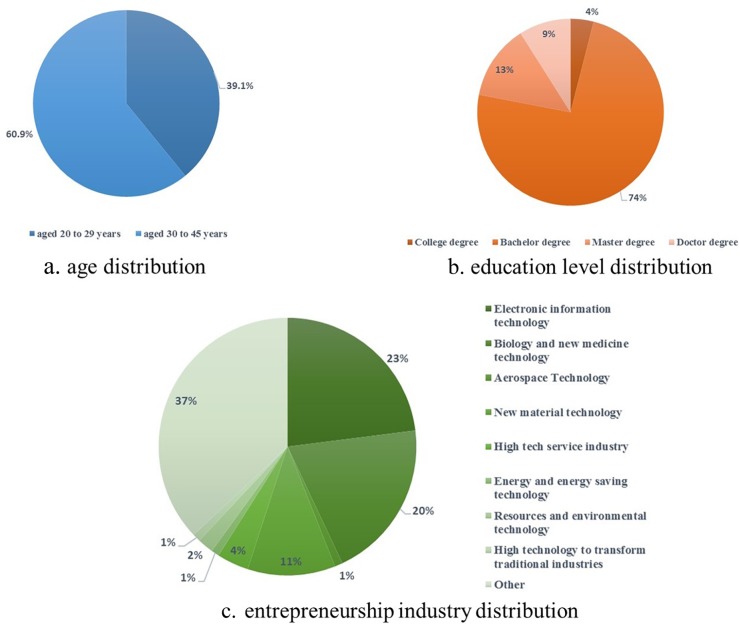
Personal characteristics of samples.

The investigated personal characteristics of the entrepreneurial followers include three aspects—namely, age, education level, and the entrepreneurship industry they work in. The descriptive graphs are shown in Figure 1. It can be seen that the number of entrepreneurial followers between the age of 30 and 45 years occupy 60.9 percent of the total entrepreneurial followers studied. It can also be seen that there is an inverse U-shape relationship between educational background and entrepreneurship. In addition, we found that new technology industries are more attractive for entrepreneurial followers. A crossover analysis between the entrepreneurship industry and education levels was also implemented. The results are displayed in Table 2.

**Table 2:** Results of the crossover analysis between the entrepreneurship industry and education levels.

			Education level				Total
		
			College	Bachelor	Master	Doctor	
Industry	Electronic information technology	Frequency	0	8	16	22	46
		Percentage of industry	0.0%	17.4%	34.8%	47.8%	100%
		Percentage of education level	0.0%	12.9%	32.0%	25.6%	23%
		Percentage of total	0.0%	4.0%	8.0%	11.0%	23%
	Biology and new medicine technology	Frequency	2	16	2	20	40
		Percentage of industry	5.0%	40.0%	5.0%	50.0%	100%
		Percentage of education level	100%	25.8%	4.0%	23.3%	20%
		Percentage of total	1.0%	8.0%	1.0%	10.0%	20%
	Aerospace technology	Frequency	0	2	0	0	2
		Percentage of industry	0.0%	100%	0.0%	0.0%	100%
		Percentage of education level	0.0%	3.2%	0.0%	0.0%	1%
		Percentage of total	0.0%	1.0%	0.0%	0.0%	1%
	New material technology	Frequency	0	8	2	12	22
		Percentage of industry	0.0%	36.4%	9.1%	54.5%	100%
		Percentage of education level	0.0%	12.9%	4.0%	14.0%	11%
		Percentage of total	0.0%	4.0%	1.0%	6.0%	11%
	High-tech service	Frequency	0	4	2	2	8
		Percentage of industry	0.0%	50.0%	25.0%	25.0%	100%
		Percentage of education level	0.0%	6.5%	4.0%	2.3%	4%
		Percentage of total	0.0%	2.0%	1.0%	1.0%	4%
	Energy and energy saving technology	Frequency	0	0	0	2	2
		Percentage of industry	0.0%	0.0%	0.0%	100%	100%
		Percentage of education level	0.0%	0.0%	0.0%	2.3%	1%
		Percentage of total	0.0%	0.0%	0.0%	1.0%	1%
	Resources and environmental technology	Frequency	0	0	2	2	4
		Percentage of industry	0.0%	0.0%	50.0%	50.0%	100%
		Percentage of education level	0.0%	0.0%	4.0%	2.3%	2%
		Percentage of total	0.0%	0.0%	1.0%	1.0%	2%
	High technology to transform traditional industries	Frequency	0	0	0	2	2
		Percentage of industry	0.0%	0.0%	0.0%	100%	100%
		Percentage of education level	0.0%	0.0%	0.0%	2.3%	1%
		Percentage of total	0.0%	0.0%	0.0%	1.0%	1%
	Other	Frequency	0	24	26	24	74
		Percentage of industry	0.0%	32.4%	35.1%	32.4%	100%
		Percentage of education level	0.0%	38.7%	52.0%	27.9%	37%
		Percentage of total	0.0%	12.0%	13.0%	12.0%	37%
Total		Frequency	2	62	50	86	200
		Percentage of industry	1.0%	31.0%	25.0%	43.0%	100%
		Percentage of education level	100%	100.0%	100%	100%	100%
		Percentage of total	1.0%	31.0%	25.0%	43.0%	100%


According to Table 2, entrepreneurial followers with a doctoral degree in the electronic information technology industry have the maximum frequency, which is 22. In addition, entrepreneurial followers with college degrees have the minimum frequency. Despite other industries, the electronic information technology, biology and new medicine technology, and new material technology industries have high frequencies, which is mainly due to the regional and national policies of recent years. Moreover, according to the investigation results of entrepreneurial industries chosen by entrepreneurial followers, entrepreneurial activities are concentrated in basic research and applied research, while activities such as technology product marketing and technology work management are not universally popular, despite impacting on the development of entrepreneurial followers.

**FIGURE 2:**
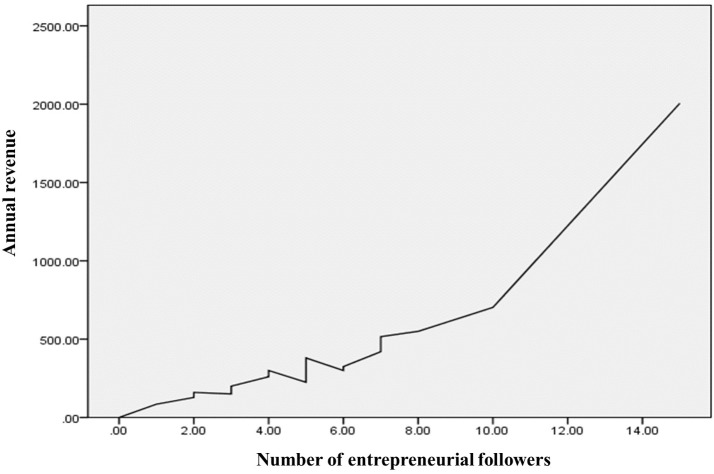
Linear correlation graph showing the number of entrepreneurial followers and the annual revenues of the sampled companies.

In addition, some indirectly evidence proving the contribution of entrepreneurial followers on the development of entrepreneurial companies has been provide by some existing papers (Ensley et al., 2006; Patterson et al., 2008). In order to further verify this conclusion through real data in China, a crossover analysis between the number of entrepreneurial followers and the annual revenues of the researched companies was implemented, shown in Table 3 and Figure 2. It can be seen that there is a piece-wise increasing function between these two elements; this is particularly noticeable when the number of entrepreneurial followers reached 10 and the marginal revenue of the company then began to accelerate.

**Table 3:** Results of the crossover analysis between the number of entrepreneurial followers and the annual revenues of the researched companies.

			Annual revenue (unit: RMB million)				Total
		
			1 to 2	2 to 4	4 to 10	Over 10	
Number of entrepreneurial followers	Less than 5	Count	3	1	0	0	4
		% of Total	15.0%	5.0%	0.0%	0.0%	20.0%
	5 to 10	Count	4	6	4	0	14
		% of Total	20.0%	30.0%	20.0%	0.0%	70.0%
	Over 10	Count	0	0	1	1	2
		% of Total	0.0%	0.0%	5.0%	5.0%	10.0%
Total		Count	7	7	5	1	20
		% of Total	35.0%	35.0%	25.0%	5.0%	100.0%


### The Need for Entrepreneurial Followers at the Start-Up Stage

#### Factors That Influence Entrepreneurship

In the interview, 11 potential factors that influence entrepreneurship were considered, and we asked the entrepreneurial followers to choose the most important ones. The responding results are shown in Table 4.

**Table 4:** Responding results of potential factors that influence entrepreneurship.

Factors	Responses		Percent of cases
		
	*N*	Percent	Percent
Scientific and technological achievements or patents	2	10.00%	2.56%
Work experience and interpersonal relationships	9	45.00%	11.54%
Regional entrepreneurship service systems	5	25.00%	6.41%
Superior geographical resources	0	0.00%	0.00%
High levels of social capital	12	60.00%	15.38%
Sufficient funds	11	55.00%	14.10%
Business operation model	2	10.00%	2.56%
High-quality entrepreneurial opportunities and projects	12	60.00%	15.38%
Highly cooperative teams	12	60.00%	15.38%
Suitable market opportunities	8	40.00%	10.26%
Marketing	5	25.00%	6.41%
Total	78	390.00%	100.00%


It can be seen from Table 4 that only 2.56% of entrepreneurial followers suggested that scientific and technological achievements or patents should be considered as important factors for entrepreneurship. This is due to the fact that, compared to entrepreneurial leaders, followers always have less of a future-focused vision. This finding is in accordance with some previous studies (Hellmann, 2007; McCarthy et al., 2010).

In addition, about 15.38% of followers viewed high levels of social capital, good entrepreneurial opportunities and projects, and highly cooperative teams as vital foundations of entrepreneurship. It seems that entrepreneurial followers believe social capital to be more important than sufficient funds. According to Kim and Aldrich (2005), social capital could be defined as the resources available to people through their social connections. They suggested that social capital is a natural characteristic that plays a preconditioned role in financial support for entrepreneurs. This phenomenon is common in the Chinese *guanxi* (social relationship) network, especially for start-up ventures (Batjargal, 2007). With respect to the high-quality entrepreneurial opportunities and projects, McCarthy et al. (2010) provided some evidence to support the consistency of top-down decision-making techniques through the strict hierarchy of entrepreneurial teams. This results in little room for followers to engage. In other words, it seems that instead of thinking about future plans, entrepreneurial followers are more concerned with how to effectively fulfill the tasks assigned to them by their leaders.

Additionally, our results showed that only 2.56% of followers considered the business operation model an important factor for entrepreneurship; this confirms the proposition suggested by Cooney (2005): That the core idea of entrepreneurship has often been created before the formation of the team. Finally, entrepreneurial followers believe that an effective team can achieve more than one person working alone. As Forsström-Tuominen et al. (2017) stated, teams have greater power in encouraging individuals to exchange ideas and create a much stronger impetus for entrepreneurship that is related to both social and psychological aspects. This could be viewed as central to triggering and initiating entrepreneurship.

#### Difficulties Faced by Entrepreneurial Followers in the Start-Up Stage

In the interview, we surveyed the potential difficulties faced by entrepreneurial followers in the start-up stage of entrepreneurship. The results are displayed in Table 5.

**Table 5:** Results of difficulties in the start-up stage of entrepreneurship.

Factors	Responses		Percent of cases
		
	*N*	Percent	Percent
Low wages	26	23.01%	26.0%
Poor housing	1	0.88%	1.0%
Difficulty in children’s education and employment	5	4.42%	5.0%
Difficulty in spouse’s transfer and employment	2	1.77%	2.0%
Difficulty in furthering personal development	2	1.77%	2.0%
Difficulty in researching results transformation	44	38.94%	44.0%
Difficulty in title appraisal	5	4.42%	5.0%
Difficulty in mobility	1	0.88%	1.0%
Difficulty in academic communication	11	9.73%	11.0%
Others	16	14.16%	16.0%


It can be noticed from Table 5 that 44% of entrepreneurial followers believe that the difficulty in transforming research results are urgent problems in entrepreneurship, and 26% suggested that low salaries also present considerable difficulties. These findings indicate that, unlike foreign (Patterson et al., 2008; McCarthy et al., 2010) and Chinese (Wang et al., 2013; Sochett and Daneman, 2014) entrepreneurial leaders, who focus more on entrepreneurial success and enterprise development, entrepreneurial followers focus more on income expectation and personal development. This finding may also directly reflect the main weaknesses in the current Chinese cultivation system for entrepreneurial followers.

In general, the construction of an effective and comprehensive entrepreneurship cultivation system could be viewed as an important factor in attracting more entrepreneurship teams and fostering more successful entrepreneurial firms. Thus, it is necessary to accurately understand the existing problems, which should be solved in a targeted manner. Despite the valuable findings that focus on the improvement of entrepreneurship cultivation for entrepreneurial leaders (e.g., Okudan and Rzasa, 2006; Mckonesweet et al., 2011) and entrepreneurs as a whole (e.g., Rasmussen and Sørheim, 2006; Henry and Treanor, 2010; Graevenitz et al., 2010; Li et al., 2013; Tian et al., 2016), the number of studies related to entrepreneurial followers seems to be much fewer. However, the abilities of followers and the introduction of more followers with high abilities are also indispensable elements that determine the success of entrepreneurship. Therefore, we surveyed the interviewees regarding the difficulties of the cultivation mechanism system during the early stages of entrepreneurship. The responding results are shown in Table 6.

**Table 6:** Results of the difficulties in the entrepreneurship cultivation system at the start-up stage.

Factor	First	Second	Third
Difficulty in training mechanisms	45.00%	5.00%	15.79%
Difficulty in distribution incentive mechanisms	20.00%	35.00%	10.53%
Difficulty in selection and appointment mechanisms	10.00%	20.00%	10.53%
Difficulty in talent flow mechanisms	5.00%	15.00%	5.26%
Difficulty in talent evaluation mechanisms	15.00%	10.00%	21.05%
Difficulty in guarantee mechanisms	5.00%	15.00%	36.84%
Others	0.00%	0.00%	0.00%


It can be seen from the data in Table 6 that 45% of respondents ranked the talent training mechanism as the primary difficulty in the cultivation process, and 20 and 35% ranked the difficulty in distribution incentive mechanism in first and second place, respectively. In addition, the talent evaluation mechanism was, respectively ranked in first and second place by 15 and 10% of interviewees. These findings further developed and enriched our previous discoveries. It seems that entrepreneurial followers place more importance on their personal development than income expectation. Moreover, this finding indicates that the current Chinese entrepreneurship cultivation system has overlooked the training of entrepreneurial followers to a large extent.

#### Improvements for Entrepreneurship Cultivation at the Start-Up Stage

Based on the surveyed results of Table 6, we also asked the entrepreneurial followers to propose some improvements to the entrepreneurship cultivation system, shown in Table 7.

**Table 7:** Results for the suggested improvement of the entrepreneurship cultivation system at the start-up stage.

Factor	First	Second	Third
Cultivate and enhance local talents	39.13%	4.35%	0.00%
Introduce foreign and overseas students	4.35%	4.35%	14.29%
Improve and implement the government talent work system	8.70%	8.70%	14.29%
Introduce incentive policies for all types of talents at all levels	34.78%	17.39%	9.52%
Optimize talent employment mechanisms	0.00%	26.09%	14.29%
Develop intermediary talent service agencies	0.00%	4.35%	4.76%
Create a good environment for growth of talent	8.70%	34.78%	19.05%
Encourage talents to undertake government, science, and technology projects	4.35%	0.00%	23.81%
Others	0.00%	0.00%	0.00%


According to Table 7, 39.13% of entrepreneurial followers believed that the government should first cultivate and enhance local talents. This interesting finding reflects the traditional Chinese belief in hometown identity, and this is especially common in Chengdu, where the culture is both receptive and inclusive. From a policy perspective, a more important concern is that there is a huge gap between the supporting force of the government for local versus returned talents in China. The latter always receives better salary and cultivation opportunities when compared to the former. This phenomenon is also common in Chinese universities and scientific institutions. As seen in Normile (2018), the only reason for a United States ecologist’s move from the University of Hong Kong (HKU) to the Southern University of Science and Technology is because he can earn 40 times more than what he is currently receiving in research support at HKU. This kind of priority is due to the lack of high-level innovative people in China. However, with the sustainable development of the Chinese economy and the growth of national power, the international status of Chinese cultural and technological forces has increased. As a result, a considerable decrease has emerged in this gap between local and returned talents, which could be viewed as highly important for potential entrepreneurial leaders or followers in the future (e.g., Jack Ma and Jun Lei are world-famous entrepreneurs who are both local talents). Thus, we claim that although significant achievements have been reached, keeping these relatively unfair and inappropriate investment and policy guidelines may harm the entrepreneurial enthusiasm of local entrepreneurs.

In addition, this high interest in the cultivation of local talents and the finding that 34.78% of entrepreneurial followers ranked a good environment for talent growth in second place are findings that are consistent with our previous argument: Entrepreneurial followers are more concerned with their personal development than treatment conditions. Moreover, 34.78% of interviewees ranked the introduction of various types of incentive policies in first place, which indicates an urgent need to raise incentives for entrepreneurial followers in the cultivation process during the start-up stage. This finding is in accordance with McCarthy et al. (2010), who proposed that incentives played a dominant role in motivating entrepreneurial employees to carry out more entrepreneurial activities. However, as the primary agent of cultivation and investment in entrepreneurship, the Chinese government is often profit- and achievement-oriented. This leads to a considerable ignorance of early entrepreneurship projects with lower profits and entrepreneurial followers with fewer accomplishments. This weakness may inevitably lead to a reduction in the possibility of creating potentially outstanding entrepreneurs and start-ups.

## Discussion

Previous studies have provided some indirectly evidence to prove the important role played by entrepreneurial followers in the development of entrepreneurial companies (Ensley et al., 2006; Patterson et al., 2008). In view of this, on one hand, Ilies et al. (2005) suggested that a high-quality leader-follower relationship could provide more open communication and strong value congruence. On the other hand, existing studies also stated that the followers’ pursuing behavior of self-interest is conducive to the development of entrepreneurship at the start-up stage (Ensley et al., 2006). In order to obtain higher profits, they would contribute their biggest strength to the growth of companies. Moreover, this behavior indirectly reflects the speculative psychology commonly existing in Chinese entrepreneurial followers, which is mainly caused by their poor conditions and survival pressures.

In addition, seen from a long-term perspective, different to leaders, followers are always faced with the risk of being dismissed, especially when companies are in financing difficulties (Zhang et al., 2018). In addition, due to the extensive financial pressure of entrepreneurship and their high levels of education, followers’ entrepreneurial activities are often profit-oriented and research-oriented. However, the Chinese government and investment institutions often have high risk-aversion characteristics. In most cases, only the entrepreneurial projects that have been successful are financially supported. Finally, they may become victims in the development process of companies. To some extent, this argument is consistent with our findings that entrepreneurial followers focus more on income expectation and personal development. However, in addition to the short-term gains brought by them, there are also some long-term risks. According to Zhang et al. (2018), 50.4% of registered firms failed within 5 to 10 years from 2008 to 2012 in China, and this failure rate is continuing to grow. They also suggested that the inclination to pursue short-term interests should be considered as one of main reasons for the failure of entrepreneurship. Although there are various factors contributed to this high failure rate and short survival time of Chinese entrepreneurial companies, the weaknesses in the current Chinese cultivation system for entrepreneurial followers to a large extent plays a vital role (Tian et al., 2016). Thus, it is of great significance to improve the cultivation of entrepreneurial followers by finding out what it is that they really need.

In order to realize this purpose, three main areas were studied in this paper—namely, influencing factors for entrepreneurship, difficulties faced by entrepreneurial followers at the start-up stage, and suggestions for the improvement of entrepreneurship cultivation systems at the start-up stage. The results show that high levels of social capital, good entrepreneurial opportunities and projects, and highly cooperative teams were viewed as vital foundations of entrepreneurship by samples. This finding indicates that Chinese entrepreneurial followers pay more attention to income expectation and personal development rather than supports that contribute to the development of companies. In addition, they are also concerned about the *guanxi* (social relationship) network in entrepreneurship activities, which is a unique phenomenon in China.

Moreover, nearly half of the studied entrepreneurial followers held that the difficulty in transforming research results was the most serious problem in entrepreneurship in China, and more than a fifth of those believed that low salaries present considerable difficulties as well. With respect to the difficulties faced by Chinese entrepreneurial followers in the cultivation process, talent training mechanism was considered as the primary one. It seems that rather than income expectation, Chinese entrepreneurial followers place more importance on their personal development; in most cases, higher capability often equates to higher income. Furthermore, as nearly 40% of entrepreneurial followers suggested that the Chinese government should pay more attention to the cultivation of local talents, an unbalance and unfair distribution of supports between local and returned talents seems to be proved. Additionally, we also found that the lack of incentive mechanism is also an existing problem. These points reflect some dominant problems of the current entrepreneurship cultivation system in China.

### Theoretical Implications

Our analysis makes some important contributions to current knowledge. First, given the importance of entrepreneurial followers for the success of entrepreneurship (Ensley et al., 2006; Patterson et al., 2008), our research firstly defined this special group as the crucial members (or crucial employees) of a specific entrepreneurial team and do not include the leader or normal employees. Entrepreneurial followers have highly consistent interests or beliefs with leaders and are generally characterized initiative, activeness, enthusiasm, and creativity. On the basis of following time, Entrepreneurial followers can be categorized into two groups: long-term and short-term, in which the former shows greater loyalty and autonomy, has more interest or belief foundation with the leaders, and has more influence on the shape of leaders’ behaviors. In addition, we also extended the existing related literature by proposing that entrepreneurial followers pay more attention to the benefits that can be gained through participating in entrepreneurial activities at the start-up stage. In a more specific sense, the income expectation and personal development opportunities could be viewed as the two main concerns of entrepreneurial followers, thus providing a more comprehensive and detailed understanding of this special group who have been commonly ignored by previous studies. In addition, we also found that these factors lead to a healthy, mutual, and complementary relationship that contributes to the development of entrepreneurship.

Second, unlike existing research, which only focused on entrepreneurship cultivation for entrepreneurial leaders or entrepreneurs as a whole (Dabic et al., 2016; Din et al., 2016), the present study investigated the needs of entrepreneurial followers in the cultivation process. Based on the results of this empirical study, we suggested that the Chinese government should pay more attention to the cultivation of entrepreneurial followers, especially local ones, by publishing more rational and comprehensive policies that target entrepreneurial companies at the start-up stage. This study thus fills a gap in the literature of entrepreneurship cultivation for entrepreneurial followers.

Third, through multiple investigation angles and various experimental methods, we tested the needs of entrepreneurial followers in entrepreneurship cultivation from the start-up stage. Our findings focused on the factors that influence entrepreneurship, the current difficulties faced by followers, and improvement suggestions for entrepreneurial cultivation, thus making our conclusion more credible and forming a reasonable, logical relationship to enable related researchers to comprehensively understand this point. Thus, this study contributes to the literature of entrepreneurship cultivation and entrepreneurial followers.

### Practical Implications

The present paper has some practical implications, as it can help policymakers, institutional investors, entrepreneurial leaders design and carry out related measures to improve the current entrepreneurship cultivation system in China and abroad. First, material incentives for the basic conditions of entrepreneurial followers should be established in order to provide them with better living and service environments. Second, the time and space advantages for cooperation between industry and university research as well as fair investment and financing systems should be built though institutional incentives. This would strengthen the technological beliefs and innovative pursuits of entrepreneurial followers. Third, traditional culture should be pertinently integrated into the entrepreneurship education system in order to enhance entrepreneurship awareness and the capacity of entrepreneurial followers.

### Limitations and Future Research Suggestions

However, there are still some limitations to the present paper:

I. This study only focused on the entrepreneurial followers in the Tianfu New Area, which limited the generality of our findings. Future studies should consider a larger sample size of multiple organizations and countries. This will make the results more representative and specific.
II. This paper only studied entrepreneurial companies at the start-up stage due to their relatively significant representativeness. However, the cultivation process is also extremely important for entrepreneurial companies in various other stages. Thus, for a more comprehensive perspective, future studies should pay attention to entrepreneurial enterprises at different growth stages.

## Conclusion

The main purpose of this paper was to answer the question that what do entrepreneurial followers really need in entrepreneurship cultivation at the start-up stage. Based on the results, following conclusions could be gained. First, high levels of social capital, good entrepreneurial opportunities and projects, and highly cooperative teams were viewed as the most important factors for entrepreneurship by entrepreneurial followers in China. Second, entrepreneurial followers considered the difficulty in transforming research results, shortage in talent training mechanism, and low salaries as main difficulties in the entrepreneurship cultivation at the start-up stage. Third, most samples believed that the government should first cultivate and enhance local talents.

These findings indicate that entrepreneurial followers in China pay more attention to their personal development than income expectation, and the social relationship in entrepreneurship activities is also an important concern. In addition, it should be noted that there is a gap between the supporting force of the government for local versus returned talents, and a lack of incentive mechanism in China.

Therefore, we claimed that the Chinese government should delivery more rational policies focusing on the cultivation of entrepreneurial followers, especially for local ones. Moreover, various types of incentive policies and good environments for talent growth were also considered as the aspects to be improved in future policies.

## Ethics Statement

This study was carried out in accordance with the recommendations of ethic guidelines, Ethic Committee of Beijing Jiaotong University with written informed consent from all subjects. All subjects gave written informed consent in accordance with the Declaration of Helsinki. The protocol was approved by the Ethic Committee of Beijing Jiaotong University.

## Author Contributions

XD, CT, and DT provided substantial contributions to the research conception and design. XD and YL analyzed and interpreted the data. XD, CT, and DT wrote the manuscript and provided critical revisions of the manuscript.

## Conflict of Interest Statement

The authors declare that the research was conducted in the absence of any commercial or financial relationships that could be construed as a potential conflict of interest.

## APPENDIX

**Table A1:** Introduces the interview topic guide for semi-structured interview conducted in the present paper.

**Introduction to Interview** Thank you for agreeing to participate in this interview. Your responses will be kept completely confidential. The aim of this interview is to discuss your viewpoints and experiences relevant to the entrepreneurship cultivation at the start-up stage. There are no “right” or “wrong” answers, and we hoped that you could give us your own ideas. Now, I am going to start the interview.
**Begin Interview** (1) What factors do you think will influence the entrepreneurship at the start-up stage? Why? Prompts • Could you provide some real examples? • Are there some special issues in China?
(2) Have you experienced some difficulties at the start-up stage of entrepreneurship? Prompts • Could you provide some details? • Do you think these difficulties are universal in current China?
(3) Have you experienced some difficulties in the entrepreneurship cultivation at the start-up stage? Prompts • Could you give me some reasons for these difficulties from your own perspective? • Do you think these difficulties are universal in current China?
(4) Could you provide some suggestions for the improvement of the entrepreneurship cultivation system at the start-up stage? Prompts • Why do you think these suggestions are useful? • Could you connect these suggestions to your above mentioned difficulties?
**Post Interview** Really thank you for participating in this interview. No more questions about this topic. However, I would like to give you an opportunity to say anything else about the entrepreneurship cultivation at the start-up stage.

**Table A2:** Presents some questions contained by the survey.

Questions
**1 Please choose the potential influencing factors of the entrepreneurship at the start-up stage**
(a) Scientific and technological achievements or patents (b) Work experience and interpersonal relationships (c) Regional entrepreneurship service systems (d) Superior geographical resources (e) High levels of social capita (f) Sufficient funds (g) Business operation model (h) High-quality entrepreneurial opportunities and projects (i) Highly cooperative teams (j) Suitable market opportunities (k) Marketing
**2 Please choose the difficulties you have experienced at the start-up stage of entrepreneurship**
(a) Low wages (b) Poor housing (c) Difficulty in children’s education and employment (d) Difficulty in spouse’s transfer and employment (e) Difficulty in furthering personal development (f) Difficulty in researching results transformation (g) Difficulty in title appraisal (h) Difficulty in mobility (i) Difficulty in academic communication (j) Others
**3 Please choose the difficulties you have experienced in the entrepreneurship cultivation at the start-up stage?** First order___; Second order___; Third order___.
(a) Difficulty in training mechanisms (b) Difficulty in distribution incentive mechanisms (c) Difficulty in selection and appointment mechanisms (d) Difficulty in talent flow mechanisms (e) Difficulty in talent evaluation mechanisms (f) Difficulty in guarantee mechanisms (g) Others
**4 Please give some suggestions to improve the entrepreneurship cultivation system at the start-up stage.** First order___; Second order___; Third order___.
(a) Cultivate and enhance local talents (b) Introduce foreign and overseas students (c) Improve and implement the government talent work system (d) Introduce incentive policies for all types of talents at all levels (e) Optimize talent employment mechanisms (f) Develop intermediary talent service agencies (g) Create a good environment for growth of talent (h) Encourage talents to undertake government, science, and technology projects (i) Others
